# Computed tomography and magnetic resonance imaging features of primary liver perivascular epithelioid cell tumor with renal angiomyolipoma: a case report and literature review

**DOI:** 10.3389/fonc.2025.1534250

**Published:** 2025-06-18

**Authors:** Ruoling Gao, Jiaying Liu, Qingdian Cong, Zhilan Huang, Guoping Zhu, Xuan Jin, Jibo Hu

**Affiliations:** Department of Radiology, the Fourth Affiliated Hospital of School of Medicine, and International School of Medicine, International Institutes of Medicine, Zhejiang University, Yiwu, China

**Keywords:** liver, perivascular epithelioid cell tumor, PEComa, renal angiomyolipoma, imaging manifestations, CT, MRI

## Abstract

Hepatic perivascular epithelioid cell tumor (PEComa) is an extremely rare mesenchymal tumor. The disease has few specific clinical symptoms and imaging manifestations, making its accurate diagnosis an intractable clinical challenge. This is a report of a female patient diagnosed with lesions and a mass in the left lobe of the liver. The computed tomography (CT) findings showed that the scan without a contrast agent had a slightly low density, and significant enhancement was seen in the arterial phase of the enhanced scan, with numerous tortuous arteries. The enhancement slightly decreased in the equilibrium phase and the delayed phase. The magnetic resonance imaging (MRI) findings were low signal for T1-weighted images (T1WI) and high signal for T2-weighted images (T2WI), and the enhancement pattern of the enhanced scan was similar to that of CT. Subsequently, the patient underwent surgical excision to remove the tumor. Based on the positive immunohistochemical staining for human melanoma black 45 (HMB45), smooth muscle actin (SMA), and melanin-A (Melan-A), a definitive diagnosis was made. Given that the pathological findings indicate low-grade malignancy, regular follow-up should be conducted. The patient presented with multiple fatty lesions in both kidneys, with the larger one located in the lower part of the left kidney, which was eventually confirmed as angiomyolipoma through surgical pathology. A literature review was carried out on the clinical features and imaging findings of the hepatic perivascular epithelioma, and cases with liver PEComa and kidney AML were described.

## Introduction

Perivascular epithelioid cell tumor (PEComa) is a mesenchymal tumor presenting with the histologic and immunophenotypic features of perivascular epithelioid cells. It is a clinically rare condition with multiple organogenetic possibilities (1, 2). PEComas consist of several lesions including angiomyolipoma, lymphangiomyomatosis, and other lesions comprising perivascular epithelioid cells in soft tissues (3, 4). Renal angiomyolipoma is the most common type, harboring varying proportions of adipose tissue, smooth muscle cells, and abnormal thick-walled blood vessels (5). The origin of PEComa in the liver is extremely rare, with the majority of cases being benign, and only a few are malignant (2, 6).In recent years, the number of cases of malignant PEComa in the liver has increased (7). Generally, radical surgical resection has been shown to result in favorable outcomes.

In this article, we will present a pathologically confirmed case of hepatic PEComa with renal AML treated at our hospital. We retrospectively discuss the clinical, radiographic, and histologic features. To further characterize the imaging features of hepatic PEComa, we also reviewed the imaging data in the reported literature.

## Case presentation

**Figure 1:**
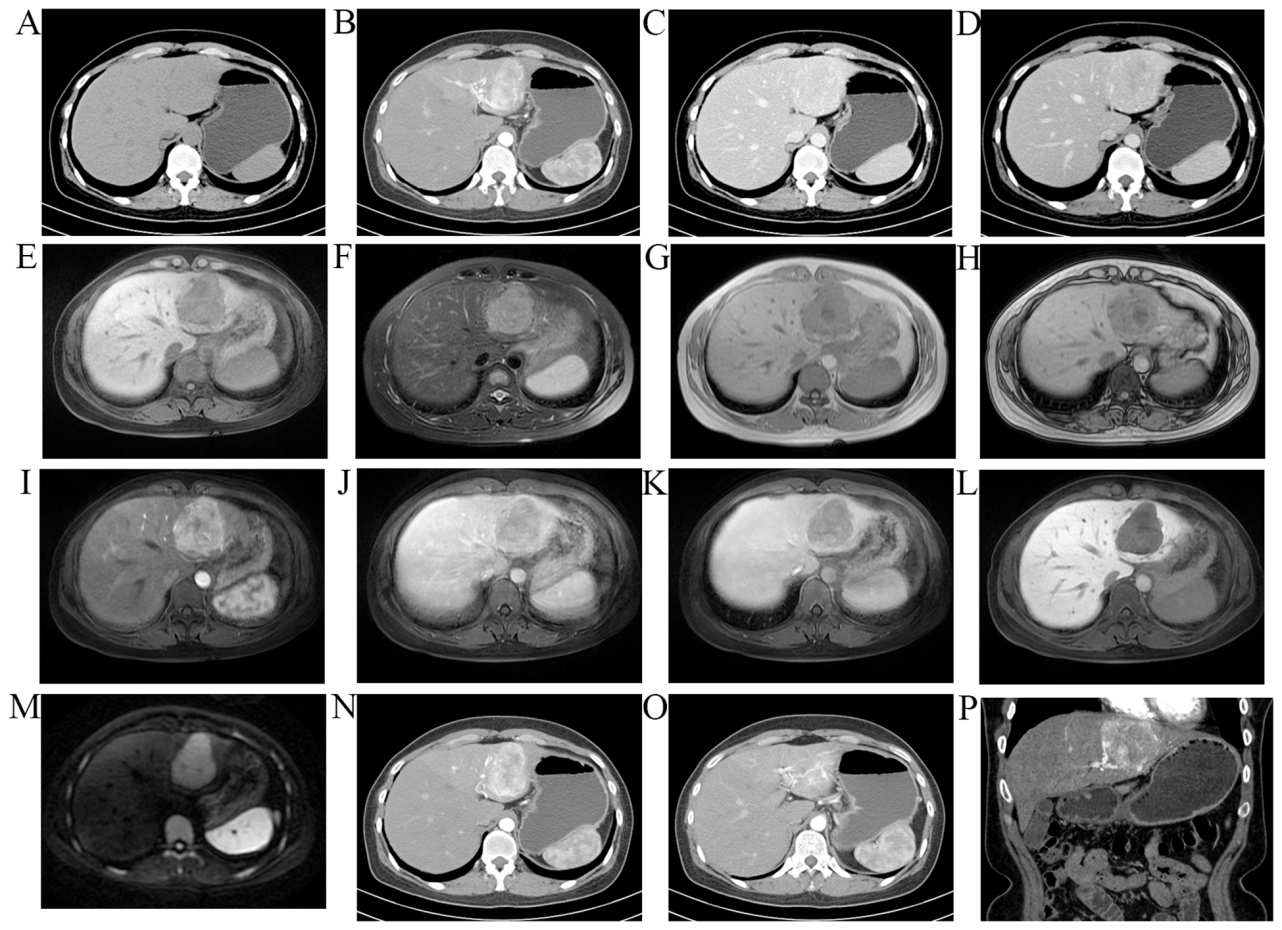
CT images of the hepatic PEComa **(A–D)**. **A**, Noncontrast CT image showing a slightly hypodense lesion in the left lobe of liver. **B**, Contrast-enhanced CT image indicating marked and heterogeneous enhancement during the arterial phase (arrow). C and D, the contrast agent is washed out during the portal and equilibrium phase (arrows). MR images of the hepatic PEComa **(E–M)**. T1WI presenting with slightly lower signals **(E)**, T2WI indicating slightly higher signals **(F)**. There was no significant signal reduction in the out-phase compared with the in-phase **(G, H)**. DWI displaying a high signal **(M)**. The lesion remained hypovascular on arterial phase (I) and portal/late venous phase **(J, K)** imaging. The contrast agent was washed out during the portal and equilibrium phase. No uptake of hepatobiliary contrast media was observed on 20-min delayed hepatobiliary phase imaging **(L)**. **(N–P)** The left hepatic artery thickened blood supply can be observed in contrast with the left hepatic lobe lesions in the enhanced arterial phase; **N, O** is transverse and P is sagittal. CT = computed tomography, PEComa = perivascular epithelioid cell tumor.

A 36-year-old female patient had a liver mass and multiple fatty lesions in both kidneys for 6 months which were detected during physical examination. At that time, the patient experienced swelling of the hands and feet but had no other discomforts. During admission, the patient had no discernable symptoms and requested further diagnosis and treatment. Imaging examination through CT revealed a mass in the left lobe of the liver, approximately 54 × 51 mm in size, with an irregular shape and a clear boundary. In the arterial phase of enhanced scanning, significant enhancement was seen, and multiple tortuous vascular shadows were detected around it. The blood supply artery was the left hepatic artery, and the portal and equilibrium phases showed equal or slightly lower density (**Figures 1A–D**). The MRI result showed a mass in the left lobe of the liver—T1WI presented with slightly lower signals, while T2WI revealed slightly higher signals—and a few mottled hyper-signals in the lesion. DWI showed a high signal, and a significantly uneven enhancement was observed at the arterial stage of Gd-enhanced images. The enhancement in the portal and delayed phases decreased significantly, and the hepatobiliary phase showed a low signal. There was no significant signal reduction in the out-phase compared with the in-phase (**Figures 1E–M**). The laboratory tests revealed normal liver function, with a-fetoprotein (AFP), carcinoembryonic antigen (CEA), and carbohydrate antigen 19–9 (CA19–9) concentrations falling within the normal range, and the patient had a history of two cesarean sections. The patient is currently in good health, with no known family history of similar conditions in parents or siblings.

**Figure 2:**
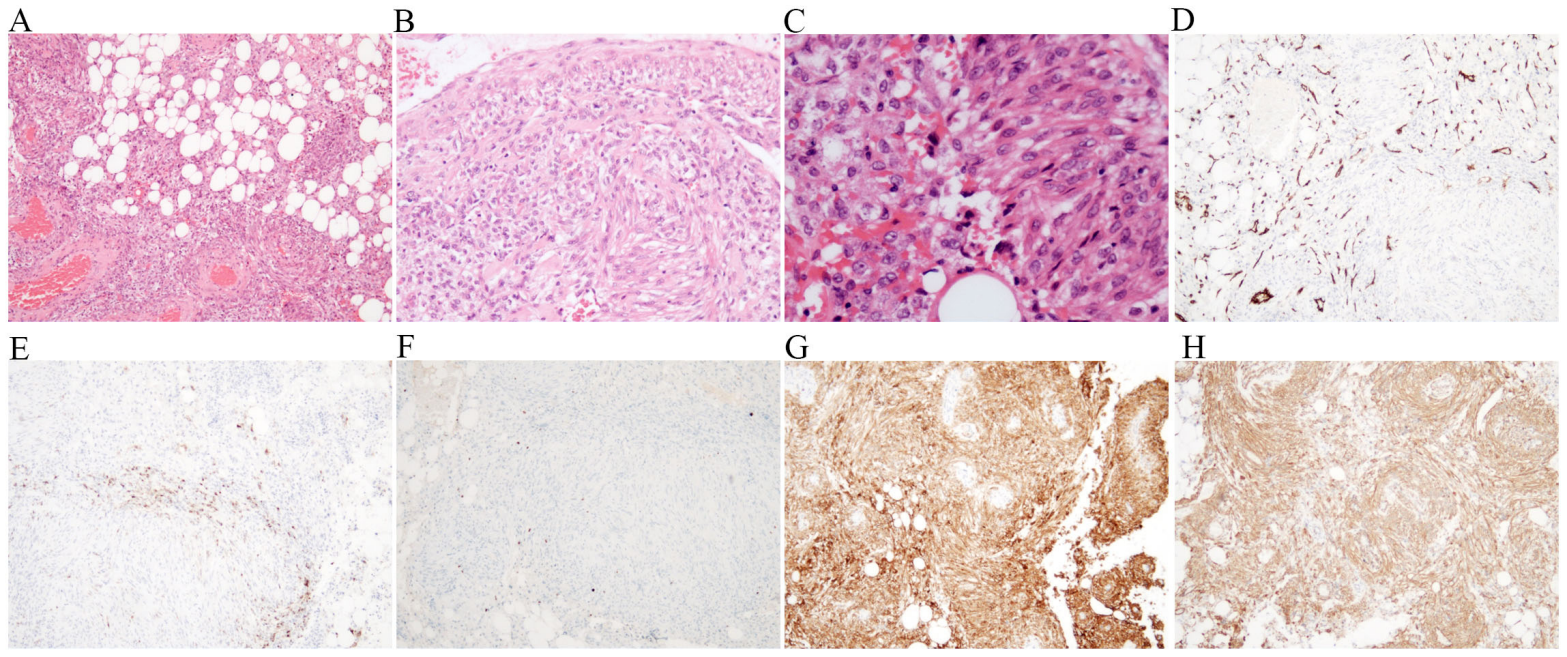
Histopathologic and immunohistochemical features of liver PEComa. Spindle-shaped cells featuring elongated cells and spindle-shaped cells. Eosinophilic cytoplasmic cells presenting transparent cytoplasm and dispersed particles (**A**, **B**: HE ×40; **C**: HE ×100; **D**: HE ×200). CD34 (vascular) was positive **(E)**. The tumor cells were focally immunoreactive for HMB45 **(F)** and diffusely immunoreactive for Melan A **(G)** and SMA **(H)**.

The patient underwent laparoscopic radical resection of left extrahepatic lobectomy and cholecystectomy under general anesthesia during hospitalization. Intraoperative exploration uncovered adhesion in the upper abdomen, without significant ascites in the abdominal cavity, and no obvious tumor implantation metastases in the greater omentum, mesentery, and pelvic cavity. A 7 × 6-cm tumor was detected in the left lateral lobe of the liver. The operation was performed successfully. A section examination of the specimen revealed that the 7 × 6-cm mass in the left extrahepatic lobe, which protruded from the liver surface, was hard in quality, and the section was grayish-white. Pathological diagnosis results confirmed the diagnosis of hepatic perivascular epithelioid cell tumor (low-grade malignancy). The immunohistochemical results were as follows: CD34 (vascular +), HMB45 (focal +), Ki-67 (1%), MelanA (+), SMA (+), SDHB (+), and S100, HepPar-1, CK19, GPC3, GATA3, CD10, PAX8, CA-IX, and CK7 were negative (**Figure 2**).

**Figure 3:**
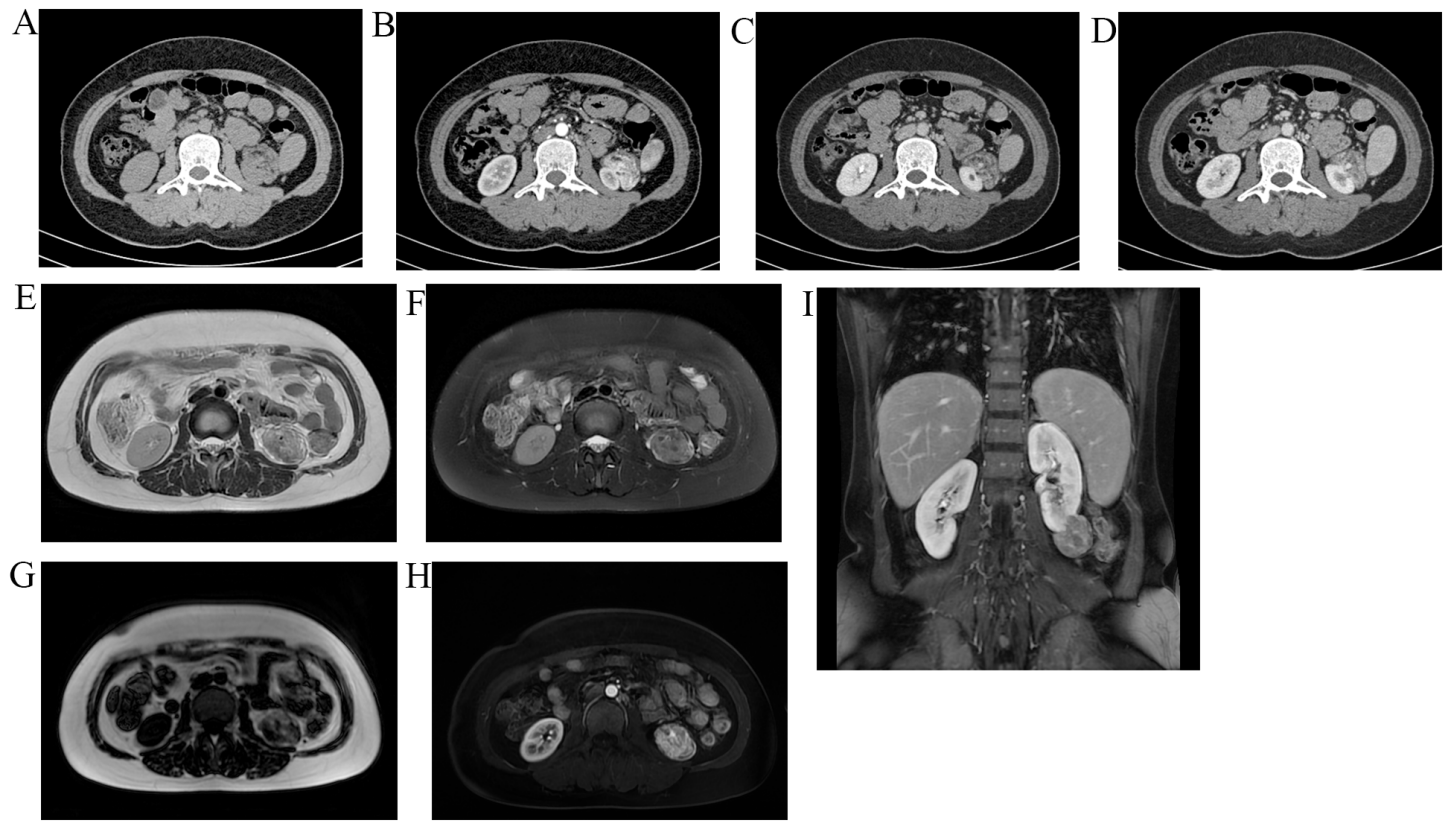
Imaging of the lesion in the lower left kidney of this female patient; **(A-D)** represent CT images. **(A)** Without contrast agents, the lesions in the CT images exhibit equal and low density. **(B)** Enhanced scans reveal significant uneven enhancement in the cortical phase. **(C, D)** Enhanced scans show a gradual decrease in enhancement in the parenchymal phase and the excretory phase. **(E-I)** are MRI images. **(E)** is T2WI without fat suppression, where the lesion presents as a non-uniform high signal. **(F)** is T2WI with fat suppression signal, and the lesion shows a high signal, with some speckled low signals within the lesion. **(G)** is the lipid phase, and numerous speckled high-signal shadows are seen in the lesion. **(H, I)** are enhanced scan images, with significantly uneven enhancement in the early stage and a decreased degree of enhancement in the late stage.

**Figure 4:**
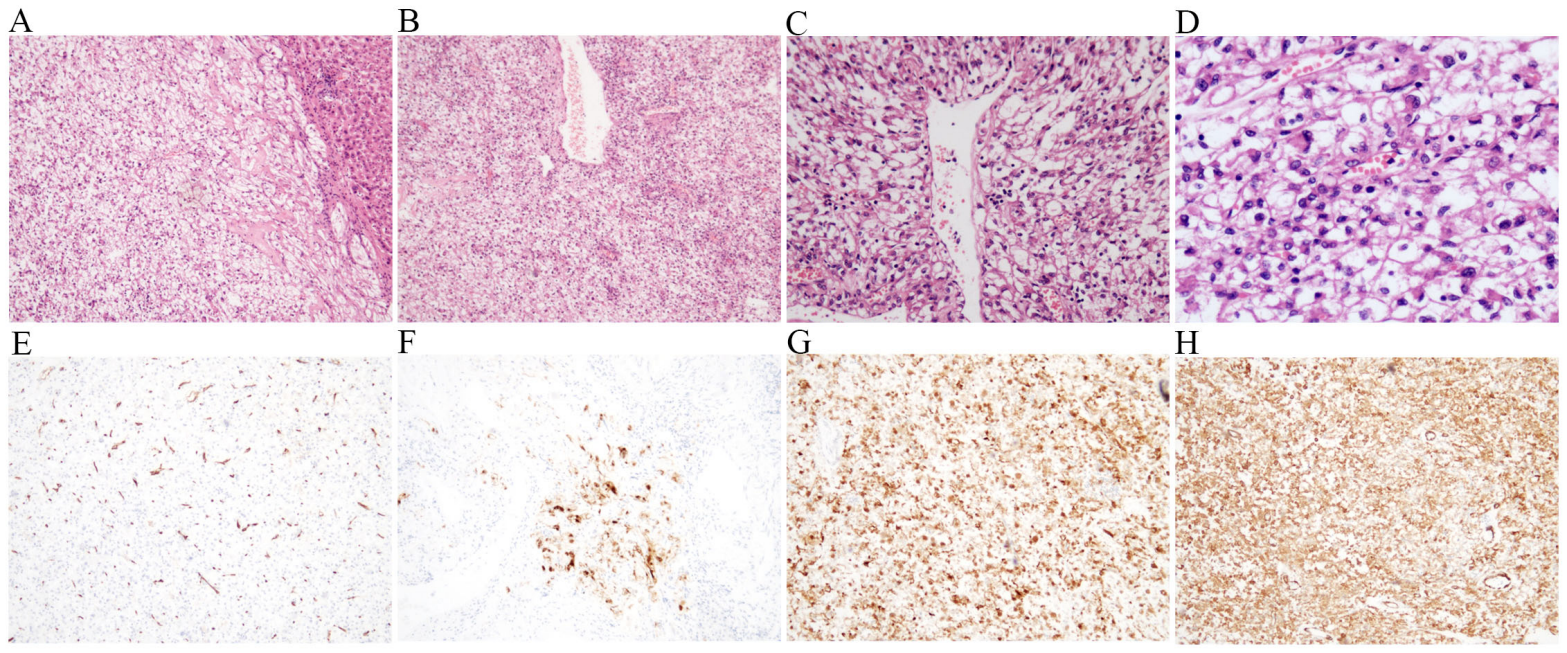
Histopathologic and immunohistochemical features of the left kidney AML. The tumor was composed of smooth muscle tissue, thick-walled blood vessels, and adipose tissue (**A**: HE ×40; **B**: HE ×100; **C**: HE ×200). **(D-H)** IHC images of the left kidney lesion: CD34 (vascular) was positive **(D)**, HMB45 was positive **(E)**, Ki-67 proliferation index was 3% **(F)**, Melan A was positive **(G)**, and SMA (smooth muscle) was positive **(H)**.

There were multiple lesions in both kidneys during the examination. In the non-contrast CT, most of the lesions were of low density in both kidneys, and the measured CT value was approximately -64 HU. The larger lesion was located in the lower part of the left kidney, measuring approximately 39 × 32 mm (**Figure 3**), with equal and low mixed density lesion in non-contrast CT. Most of the small lesions did not show significant enhancement during enhanced scan, and the lesions in the lower pole of the left kidney exhibited uneven and pronounced enhancement, and the enhancement in the parenchymal and equilibrium stages was reduced. The MRI result revealed that the signal of the out-phase was significantly lower compared with that of the in-phase, confirming the existence of fat components. In addition, speckled and patchy high-signal shadows were seen in the fat imaging, further confirming the existence of fat components. The enhanced scan revealed a tortuous vascular shadow in the larger lesion in the lower part of the left kidney. The MRI enhancement was similar to that of the enhanced CT images. Collectively, the imaging findings strongly suggested angiomyolipoma. Notably, the lower pole of the left kidney was relatively larger and exhibited tortuous blood vessels within the lesion. The urologists suspected the possibility of a tumor rupture and hemorrhage. In this case, total resection of the left kidney was proposed, creating significant anxiety in the patient. At 1 year after liver surgery, the patient was re-admitted to the hospital for resection of the larger lesion in the left kidney. On admission, the value of cytokeratin 21–1 was slightly elevated (3.21, normal range <2.08), but the results of other laboratory tests were normal. The operation was successfully completed. The pathological results confirmed the diagnosis of angiomyolipoma (AML). The immunohistochemical results were as follows: S-100 (fat +), MelanA (+), HMB45 (+), CD34 (vascular +), SMA (smooth muscle +), Desmin (partial +), and Ki-67 (+, approximately 2%); STAT6, EMA, and CD117 were negative (**Figure 4**). The larger lesion located in the left kidney was excised through surgical operation. The pathological analysis confirmed that the lesion was benign. For the smaller lesions in the remaining kidneys, the urologist recommended surveillance through periodic follow-up exams. Surgical intervention would be reconsidered upon detection of lesion enlargement or other concerning changes. Furthermore, it was considered that performing surgery on all lesions in both kidneys could result in substantial surgical trauma, potentially compromising renal function and consequently affecting the patient’s overall quality of life.

No tumor recurrence or metastasis was detected at 12 and 19 months after liver surgery (**Appendix S1**). The postoperative follow-up at 7 months included a Doppler ultrasound, which revealed no signs of recurrence in the left kidney. The coronal MRI images of the liver showed the postoperative renal status, with no significant abnormal signals detected in the surgical area (**Appendix S2**). In recent years, we have been following up on the patient through phone, during which the patient reported feeling more tired than before the surgery, but overall, it did not affect her daily work and life.

## Discussion

PEComas were first described in 1992 by Bonetti et al. (8) and subsequently confirmed as a distinct category in the World Health Organization Classification of Tumors in 2002 (9). PEComas present with several types of lesions including angiomyolipoma, lymphangiomyomatosis, clear cell “sugar” tumor of the lung, and other lesions comprising perivascular epithelioid cells in soft tissues (3, 4). According to the latest edition (5th edition) of the 2020 World Health Organization classification, the use of terms such as clear cell myomelanocytic tumor and sugar tumor of the lung is discouraged. However, epithelioid AML is considered as a synonymous term for PEComa (7). Within the hepatic PEComa spectrum, angiomyolipoma is the most prevalent tumor type. If adipose tissue is present in the lesion, MRI imaging provides clear detection, and it is only present in some typical hepatic epithelioid angiomyolipoma (HAML) (10). In some cases, even if the final diagnosis is HAML, there is no fat attenuation in the in-phase and out-phase (11, 12). In the present case, MRI imaging confirmed the absence of fatty components within the lesion while positive CD34 staining was detected, which indicated the presence of vascular components. Some cases exhibited neither fat nor vascular components, and there was no direct association with ligament structures; these were classified as PEComa—not otherwise specified (PEComa-NOS), accounting for approximately 10.3% of reported cases in the available literature (3). The most common PEComas are the ovaries, uterus, gastrointestinal tract, bladder, abdominal wall, heart, and pancreas (13). Therefore, primary liver PEComa is an extremely rare mesenchymal tumor.

Primary hepatic PEComa affects people of diverse ages, with a median age of onset at 47 years, underscoring the prevalence of this disease among the middle-aged population. The disease exhibit gender difference, being more prevalent in women than their male counterparts. Clinically, approximately half of the patients are asymptomatic during the initial stages of the disease, complicating early diagnosis. The symptoms are predominantly located in the abdominal region including abdominal pain and discomfort. Moreover, the patients may experience various non-specific systemic symptoms such as nausea, anorexia, and progressive weight loss (3). These manifestations may arise from the direct compression or invasion of surrounding tissues by the tumor as well as the potential disruption of various metabolic and nutritional status.

The boundary of primary PEComas presents with unique imaging findings; however, its imaging manifestations are not typical. In non-contrast CT, it commonly presents as lesions of equal or slightly lower density. The MRI findings reveal high signal intensity on T2WI and low signal intensity on T1WI (9, 14–16); some lesions have T1WI that is isosignaling (13). The image enhancement patterns of PEComas vary. Previous studies show that the lesions exhibit significant enhancement, with non-uniform enhancement being more prevalent (2, 6, 11, 13, 17–21). Some of the focal areas may present with cystic unenhanced areas. In CT and MRI images, these tumors are characterized by a significantly enhanced arterial phase and decreased contrast in the veno-portal and delayed phases due to the abundance of branches of the hepatic artery, resembling the features seen in hepatocellular carcinoma (HCC) (6, 19, 20, 22, 23). The supplying artery in one case was the right hepatic artery (19). This case was also diagnosed as HCC before surgery, especially in MRI, exhibiting a “fast-forward and fast-out” enhancement mode (**Figures 1E–M**). In our case, the multiple tortuous vessels surrounding the lesion were also branches of the left hepatic artery (**Figures 1N–P**). This is consistent with the blood supply pattern of HCC, making it difficult to diagnose. Therefore, the diagnosis should involve a combination of imaging tools, tumor markers, hepatitis B history, and other examinations. Harwal et al. reported that CT-enhanced arteriography showed significant and continuous enhancement (21), overlapping with the features of benign tumors such as focal nodular hyperplasia (FNH) and hemangioma. FNHs reveal a highly uniform reinforcement, while hemangiomas have a typical blood pooling appearance, both of which significantly differ from the uneven reinforcement of PEComas (20). There is an overlap with another rare solid tumor of the liver: hepatic epithelioid hemangioendothelioma (HEHE). The specific imaging features of HEHE include a target sign on T2WI imaging, represented by a white target-like sign, consisting of a high-signal-intensity core, a low-signal-intensity thin ring, and a weak high-signal-intensity halo (24). HEHE is generally multiple and subcapsular, with capsular retraction and vascular termination at the lesion edge (lollipop sign), making it different from PEComa (20). Moreover, some of the lesions exhibited a close relationship with the liver vessels, and the mass was associated with the right portal vein branch (6). One case involved prominent feeding right hepatic artery and right portal vein thrombosis (21). The case reported by Hekimoglu et al. involved the inferior vena cava (13), exhibiting the features of malignancy.

(18)F-FDG PET/CT, as a crucial examination approach for the overall assessment of tumors, can efficiently discriminate between benign and malignant tumors. Wang et al. reported that the SUVmax of the lesion was 10.02, the preoperative diagnosis was a malignant tumor, and the postoperative diagnosis was HAML (Ki-67 (7%)) (11). The lesion of the other case was non-FDG avid with an SUVmax of 1.31 (in contrast to the normal hepatic SUVmax of 1.58), and the Ki-67 labeling index was 3%. Therefore, the final diagnosis of the lesion was primary hepatic PEComa without any indications of malignancy (25). However, its accuracy needs to be further investigated.

Considering that PEComa lacks typical clinical, laboratory, and radiographic manifestations, the definitive diagnosis is achieved through histology and immunohistology. All samples in the study by Han X et al. had a positive expression of HMB45^18^, which was highly specific. Moreover, SMA and Melan-A can exhibit positive reactions in the majority of cases (2, 7, 19). Ji et al. found that 86.7% of the patients had CD34 positivity; the proportions of S-100 and Desmin positivity were less than 35%, and the positive index of Ki-67 was less than 5% in the majority of cases (26). In our case, HMB45, SMA, CD34, and Melan A were positive, and the positive index of Ki-67 was 1%. TFE3, a novel marker, is highly expressed in 14% of PEComas, indicating the presence of TFE gene rearrangement (27). HMB45 and TFE3 staining exhibit high intensity in PEComas containing TFE3 gene rearrangements, but the expression of Melan A and smooth muscle markers tends to be focal or negative (28, 29). TFE3-positive PEComas present with an aggressive biological behavior with poor prognosis. Nevertheless, existing studies suggest that TFE3-positive hepatic PEComas might be less malignant than TFE3-positive PEComas in other organs (30). These immunohistochemical staining tests not only provide robust auxiliary evidence to support the diagnosis of primary hepatic PEComa but also reveal the heterogeneity of the disease at the molecular level, expanding our understanding of this rare tumor.

In recent years, the number of cases of malignant transformation of PEComa has been on the rise (7). Therefore, identification of benign and malignant PEComas is crucial. Folpe et al. (31) established the criteria to categorize PEComa of soft tissue and gynecological origin as benign, malignant, or of uncertain malignant potential (31). The Folpe criteria proposed seven histological criteria for assessment, in which PEComa with two or more features was classified as malignant. The criteria include (1) tumor size >5 cm, (2) high nuclear grade, (3) hypercellularity, (4) mitotic rate >1/50 high-power field (HPF), (5) necrosis, (6) infiltration into the surrounding normal parenchyma, and (7) vascular invasion. Tumors that display only nuclear polymorphisms or multinucleated giant cells or are >5 cm in size are considered to have unclear malignant potential (31). In the analysis of imaging manifestations, the focus should be on the size of the tumor and whether the adjacent blood vessels and tissues are invaded. This will help to evaluate the benignity and malignancy of PEComa.

Through a PubMed search using the terms “liver PEComa and kidney AML”, we identified three similar cases. Notably, one case described a 26-year-old woman with lymphoangioleiomyomatosis (LAM) who required surgical intervention for a massive right-kidney-derived abdominal mass. The pathological diagnosis was conventional AML. Two liver tumors were formed at 8 months after the operation, which exhibited rapid growth. Following tumor resection, the patient presented with a pathological manifestation of epithelioid AML. Subsequently, multiple metastatic lung tumors were detected, accompanied with the local recurrence of liver tumors. This study found that, sirolimus, an mTOR protein inhibitor, did not inhibit the rapid growth of the tumor; complete surgical resection should be the treatment of choice (32). The second case involved a 38-year-old male who underwent right nephrectomy for a renal mass pathologically diagnosed as atypical AML. The postoperative surveillance revealed multiple left renal AMLs and a new hepatic lesion in the right lobe 10 months later, necessitating surgical resection. Based on the postoperative pathology, it was concluded that the liver mass was the site of metastatic PEComa in the primary renal lesion. At 6 months after right hepatectomy, new metastatic foci appeared in the left liver lobe and lungs of the patient (33). The third case involved a 55-year-old male with tuberous sclerosis complex (TSC) presenting with left renal AML. Although the initial treatment with the mTOR inhibitor everolimus achieved a complete response, disease recurrence manifested as a left renal mass recurrence at 2 years, followed by hepatic lesion development at 5 months thereafter. Even with continuous treatment with everolimus, liver PEComa exhibited a rapid growth, occupying the entire liver within 1 year after its first discovery. The autopsy revealed that polymorphic nuclear atypia cells had spread in the liver, kidneys, and lungs. A histopathological analysis of pre-treatment renal AML samples revealed the absence of pleomorphic cells. The subsequent clinical course suggests a possible association between mTOR inhibition and PEComa malignant progression (34). In all of the identified literature, the renal tumors were first identified, followed by liver lesions after treatment. The analysis of the pathological changes further revealed cell atypia, contributing to disease progression and metastasis, which led to a poor prognosis. Notably, the role of the mTOR inhibitors appears to be controversial, and this direction needs to be further investigated.

The patient had no significant medical history but later developed isolated swelling of the hands and feet. The CT imaging revealed synchronous liver and kidney lesions, though their exact relationship remained unclear. Considering the cumulative experience from three similar cases, the possibility of hepatic metastasis from PEComa cannot be ruled out. The routine pathological findings indicate that the lesion presents with low-grade malignancy. Furthermore, the simultaneous involvement of the liver and kidneys should be considered, given their shared origin within the PEComa family. Most importantly, regular follow-up is advocated to improve early detection and proactive management of any potential malignant tumors. Long-term follow-up with this patient will be essential to determine whether a pathological link exists between the hepatic and renal lesions.

Surgical resection is an effective therapeutic approach for the majority of cases, while interventional embolization is suitable for some cases (2). Interventional embolization may be performed for tumors with abundant blood supply. In several cases, adjuvant therapy and neoadjuvant therapy were recommended, and some patients chose to undergo follow-up (3).

## Conclusion

In clinical practice, primary hepatic PEComa is prevalent among middle-aged women. A large number of patients are asymptomatic, and when present, the main symptoms are abdominal pain, discomfort, and systemic symptoms. Lesions identified in the liver without any history of hepatitis or cirrhosis should be considered potential cases of hepatic PEComa. The presence of malignant features suggests a transformation toward malignancy. In patients with a co-occurrence of renal angiomyolipoma and hepatic lesions, which is relatively rare, it is likely that these lesions may have originated from the same source. The present case creates awareness of this condition and is expected to improve the formulation of targeted diagnostic and therapeutic strategies. Therefore, imaging physicians should familiarize with the features of this rare tumor to provide an accurate diagnosis.

## Data Availability

The original contributions presented in the study are included in the article/Supplementary Material. Further inquiries can be directed to the corresponding author.
