# Supplementary material for: Computed tomography and magnetic resonance imaging features of primary liver perivascular epithelioid cell tumor with renal angiomyolipoma: a case report and literature review
Source: Front Oncol. 2025 Jun 18;15:1534250. doi: 10.3389/fonc.2025.1534250 (PMC12213450; doi:10.3389/fonc.2025.1534250)

Appendix S1. **CT examination 12 months after liver surgery (A, B), MR examination 19 months post-liver surgery (C, D); No significant mass or abnormal enhancement is seen in the operative area.**

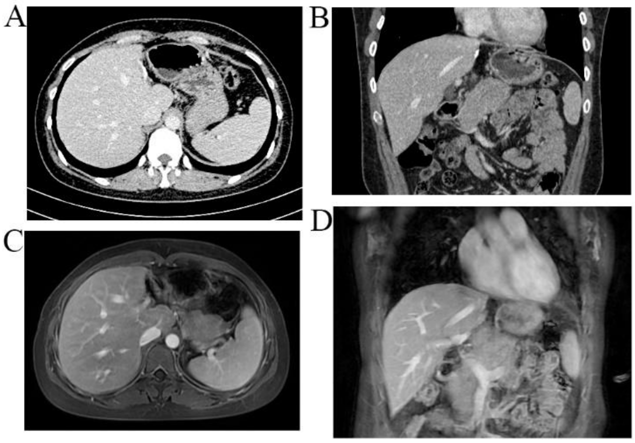

Supplement: Supplementary file 1 [file Image1.pdf]
