# Supplementary material for: Computed tomography and magnetic resonance imaging features of primary liver perivascular epithelioid cell tumor with renal angiomyolipoma: a case report and literature review
Source: Front Oncol. 2025 Jun 18;15:1534250. doi: 10.3389/fonc.2025.1534250 (PMC12213450; doi:10.3389/fonc.2025.1534250)

Appendix S2 Doppler ultrasound examination 7 months after left kidney surgery (A), Analysis of liver MRI coronal images revealed no pronounced abnormal signal shadows the surgical region (B, C); No significant mass or abnormal signal shadows were seen in the operative area.

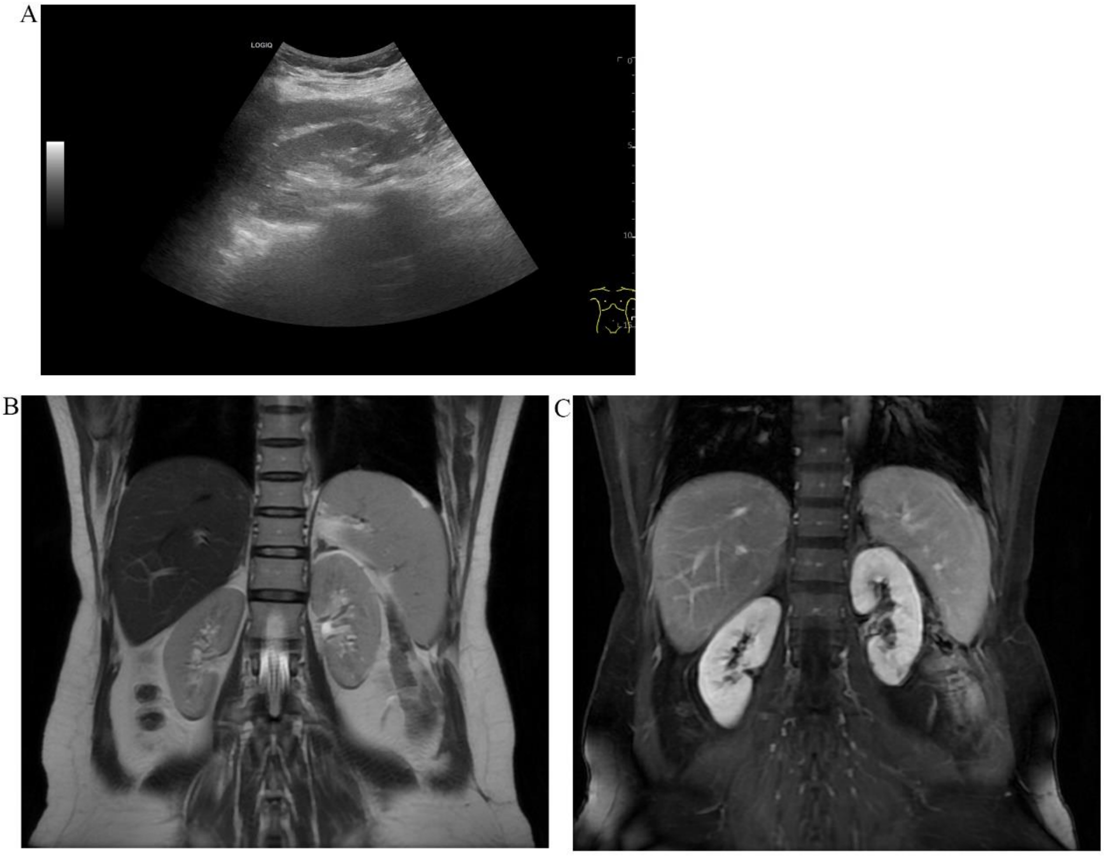

Supplement: Supplementary file 2 [file Image2.pdf]
